# EGFR/FOXO3A/LXR-α Axis Promotes Prostate Cancer Proliferation and Metastasis and Dual-Targeting LXR-α/EGFR Shows Synthetic Lethality

**DOI:** 10.3389/fonc.2020.01688

**Published:** 2020-11-02

**Authors:** Tingting Chen, Jie Xu, Weihua Fu

**Affiliations:** Department of Urology, The Second Affiliated Hospital, Third Military Medical University (Army Medical University), Chongqing, China

**Keywords:** prostate cancer, EGFR, FoxO3a, LXR-α, proliferation, metastasis

## Abstract

Prostate cancer is the second leading cause of cancer-related death in men. Early prostate cancer has a high 5-year survival rate. However, the five-year survival rate is low in progressive prostate cancer, which manifests as bone metastasis. The EGF receptor overexpression increases during disease progression and in the development of castration-resistant disease, and may be a potential therapeutic target. Liver X receptors (LXRs) are ligand-dependent nuclear receptor transcription factors and consist of two subtypes, LXR-α and LXR-β, which can inhibit tumor growth in various cancer cells. We revealed that LXR-α, but not LXR-β, was reduced in prostate cancer tissues compared with adjacent normal tissues. LXRs’ agonist GW3965 enhanced the inhibitory action of LXR-α on the proliferation and metastasis of prostate cancer cells. Furthermore, our results support the notion that LXR-α is regulated by the EGFR/AKT/FOXO3A pathway. As an EGFR inhibitor, Afatinib could weaken AKT activation and increase the expression level of FOXO3A in prostate cancer. In addition, we indicated that the combination of Afatinib and GW3965 simultaneously increased and activated LXR-α, which led to an increase of tumor suppressors, and eventually inhibited tumor progression. Therefore, the combination of EGFR inhibitor and LXRs agonist may become a potential treatment strategy for prostate cancer, especially metastatic prostate cancer.

## Introduction

Prostate cancer is the second leading cause of cancer-related death in men (1). In China, prostate cancer leads to 25,000 deaths annually, and the estimated 5-year survival rate is 54% (2, 3). Androgen receptor (AR), the first effector of testosterone action, activates certain genes involved in tumor growth and metastasis (4). Systemic therapy for advanced prostate cancer has largely focused on targeting AR. Thus, androgen deprivation therapy (ADT) is the main treatment for metastatic prostate cancer (5, 6). Unfortunately, remissions are temporary because surviving prostate cancer cells adapt to the androgen-deprived environment to form castration-resistant prostate cancer (CRPC) (7, 8). The acquisition of androgen independence may be due to the up-regulation of growth factor/receptor signaling pathways, such as the epidermal growth factor receptor (EGFR) signaling pathway (9, 10).

EGFR interacts with its ligand EGF, which leads to the activation of several downstream intracellular signaling pathways, including MEK, ERK and PI3K/AKT, and STAT3 (11). EGFR reduces tumor suppressors and activates oncogenes to promote prostate cancer progression and bone metastasis (12, 13). The expression of EGFR is correlated with the risk of recurrence and progression to hormone resistance (14). Many studies indicate that EGFR is a potential therapeutic target for prostate cancer, especially for patients with CRPC. However, some clinical trials using EGFR inhibitors alone have not found significant therapeutic effects. Afatinib, as a second-generation selective and irreversible EGFR family inhibitor, has demonstrated substantial clinical activity as a single agent in carcinomas (15–17). Recently, some studies reported that Afatinib had limited anti-tumor activity in CRPC patients (18), probably due to EGFR-targeted therapies desistance (19). Furthermore, in a phase II study, the CRPC patients showed no response to Gefitinib (another EGFR inhibitor), which showed a minimum anti-cancer activity (20). This resistance to gefitinib was perhaps due to an overactivity of the PI3K/Akt pathway in prostate cancer (21). On the contrary, the combination of EGFR inhibitors and other drugs is becoming a promising treatment for refractory prostate cancer. Inhibition of EGFR with cetuximab shows significantly improved PFS in prostate cancer patients with an overexpression of EGFR and persistent activity of PTEN (22). Thus, innovative treatment strategies are urgently needed for improving the survival of patients with prostate cancer.

Abnormal lipid metabolism plays an important role in the pathogenesis of prostate cancer. The cholesterol content of benign and cancerous human prostate glands’ tissues were essentially double that of normal prostates (23). The urinary excretion of non-esterified cholesterol (NEC) is increased in metastatic prostate cancer (24). Liver X receptors (LXRs) are ligand-dependent nuclear receptor (NR) transcription factors, including LXR-α (NR1H3) and LXR-β (NR1H2), and have been well characterized as regulators of cholesterol homeostasis and lipogenesis (25). LXR-β is distributed ubiquitously, while LXR-α is expressed highly in metabolically active cells/tissues such as the liver, macrophages, and adipose tissue (26). LXR agonists, such as T0901317 and GW3965, can not only reduce intracellular cholesterol content (27), but also activate LXRs and inhibit the proliferation of cancer cells (28–31), including prostate cancer (32, 33). In addition to enhanced *de novo* cholesterol synthesis (34), the cholesterol absorption process is also regulated by EGFR signaling activity (35). Oncogenic transformation by EGFR increases the demand for cholesterol, and EGFR signaling antagonized LXR-α effects on cholesterol homeostasis (36). Furthermore, LXRs were significantly downregulated in tumor tissues and cells with an unknown mechanism (28, 33).

Herein, we demonstrate that LXR-α, but not LXR-β, shows lower levels in prostate cancer tissues than adjacent normal tissue. High expression of LXR-α in prostate cancer cells enhances the inhibition effects of LXR agonist GW3965 on cell proliferation and invasion. In addition, our study finds that LXR-α is regulated by the EGFR/AKT/FOXO3A axis and a combination of EGFR inhibitor Afatinib and GW3965 simultaneously increases and activates LXR-α, resulting in an increase of tumor suppressors and, ultimately, tumor inhibition. Therefore, our research will provide a theoretical basis for a new therapeutic treatment of progressive prostate cancer.

## Materials and Methods

### Reagents and Cell Cultures

GW3965 (MCE, #HY-10627) and Afatinib (MCE, #HY-10261) were purchased from Selleck Chemicals, and dissolved in dimethyl sulfoxide (DMSO) (Amresco, #N182) and stored at −20°C. 22RV1, LNCaP, PC3, and DU145 cell lines were cultured at RPMI-1640 medium (Gibco, # 21870076). RWPE-1 cell line was grown in KSFM medium (Gibco, #10744019). All media were supplemented with 10% fetal bovine serum (FBS) and 1% penicillin-streptomycin. All the medium was purchased from Gibco. All cells were purchased from the Zhong Qiao Xin Zhou Biotechnology Co., Ltd. (Shanghai, China) and incubated at 37°C with 5% CO_2_.

### Cell Proliferation Assay

Cells were seeded into 96-well plates in triplicate. At different times after cell plating, cells were subjected to the Cell Counting Kit-8 (CCK-8) assay (MCE, HY-K0301), according to the manufacturer’s instructions.

### Immunoblotting (IB) and Antibodies

Western blot was performed as previously described (37). The following primary antibodies, which were purchased from Cell Signaling Technology, were used: Cleaved Caspase-3 (Asp175) (5A1E) (#9664), Cleaved Caspase-8 (Asp391) (18C8) (#9496), p21 (#2947), p27 (#3686), AKT (pan) (40D4) (#2920), Phospho-AKT (Ser473) (D9E) XP (#4060), and Bim (#2933). EGFR (#18986-1-AP), LXR-α (#14351-1-AP), LXR-β (#60345-1-Ig), CHOP (#15204-1-AP), FOXO3A (10849-1-AP), and GAPDH (#60004-1-Ig) were purchased from Proteintech. FLAG was obtained from Sigma-Aldrich (# F1804). All secondary antibodies were purchased from Cell Signaling Technology (Danvers, MA, United States).

### Plate Colony-Forming Assay

Cells were seeded into 60-mm dishes in triplicate, followed by incubation at 37°C for 14–21 days. The colonies were then fixed with 4% paraformaldehyde, stained with crystal violet, and counted (38).

### Lentivirus or Transient Transfection

For transient transfection, cells were seeded in antibiotic-free medium at 37°C for 24 h and transfected with FOXO3A siRNA or FOXO3A plasmid using lipofectamine-2000 transfection reagent (Life Technologies, Carlsbad, CA, United States) according to the manufacturer’s instructions, and treated 48 h after transfection. The lentiviruses of LXR-α overexpression were purchased at GenePharma (Shanghai, China), and cells were transfected with lentivirus according to the manufacturer’s protocol.

### Transwell Migration Assay

Transwell assay was performed as described previously (39). The cells were cultured at 37°C in a humidified atmosphere with 5% CO_2_ for 48 h. The cells below the sieve membrane were then fixed with 4% paraformaldehyde for 20 min and stained for 15 min with 0.5% crystal violet (Beyotime, China). The number of migrating cells in five fields were counted and the average number from each chamber was determined. Each experiment was conducted in triplicate.

### Quantitative Real-Time PCR

Total RNA was isolated from prostate cells using RNAiso Plus (Takara, #9108), according to the manufacturer’s instructions. Reverse transcription of the extracted RNA to corresponding complementary DNA was performed using PrimeScript RT reagent Kit with gDNA Eraser (Takara Bio, Inc., Otsu, Japan). RT-qPCR was performed with QuantiNovaTM SYBR^®^ Green PCR Kit (Qiagen GmbH, Hilden, Germany) on an Applied Biosystems 7900HT Real-Time PCR System. The following forward (F) primers and reverse (R) primers were used: LXRα (sense, GGATAGGGTTGGAGT CAGCA; antisense, GGAGCGCCTGTTACACTGTT), LXRβ (sense, GCTCAGGAG CTGATGATCCA; antisense. GCGCTTGATCCTCGTG TAG), and GAPDH (sense, ATGG TGAAGGTCGGTGTG; antisense, ACCAGTGGATGC AGGGAT). The housekeeping gene, GAPDH, was used as loading controls. Each experiment was conducted in triplicate.

### *In vivo* Xenograft Model

All animal experiments were carried out according to a protocol approved by the University Committee for Use and Care of Animals. Four- to six-week-old BALB/c athymic nude mice (nu/nu, female) were used with each experimental group, consisting of four mice. 2 × 10^6^ PC3 cells were mixed 1:1 with matrigel (BD Biocoat #354230) in a total volume of 0.2 ml and then injected subcutaneously into the right flank side of nude mice, which were randomized into four groups and treated with vehicle, GW3965 (40 mg/kg/days, every day, per gavage), Afatinib (20 mg/kg/days, every day, per gavage) and GW3965 + Afatinib, when the tumor size reached about 100 mm^3^. Mice were followed up for tumor size and body weight, and then killed after 21 days of treatment. Tumors were resected, weighed, and frozen or fixed in formalin and paraffin-embedded for immunohistochemical studies. The growth of tumors was measured twice a week and average tumor volume (TV) was calculated according to the equation: TV = (L × W^2^)/2.

### Clinical Specimens

All four pair prostate samples (including tumor tissues and adjacent tissues) were derived from biopsy samples and were finally confirmed as prostate cancer with bone metastases. The study was conducted in accordance with the Declaration of Helsinki, and the protocol was approved by the Ethics Committee of the Army Medical University (Third Military Medical University) of China. Each patient had provided written informed consent prior to surgery.

### Tissue Microarray

Tissue microarray was purchased from Servicebio (Wuhan, China), which provided chip information and completed immunohistochemical staining of the chips. IHC staining was performed on 34 paired samples (including 34 prostate tumor tissues and 34 paired adjacent tissues) for LXR detection. Furthermore, the above 34 prostate tumor tissues and another 25 prostate tumor tissues (totally 59 cases) were used to detect LXR and EGFR expression by IHC assay for further correlation analysis. The stained slides were observed under a microscope, and images were acquired and quantitatively classified based on staining intensity as described previously (40). Based on the staining intensity, we classified the samples into five groups: the weakest group (±), weak group (+), medium group (++), strong group (+++), and strongest group (++++). We defined the ±, +, and ++ groups as low-expression groups and the +++ and ++++ groups as high-expression groups.

**FIGURE 1:**
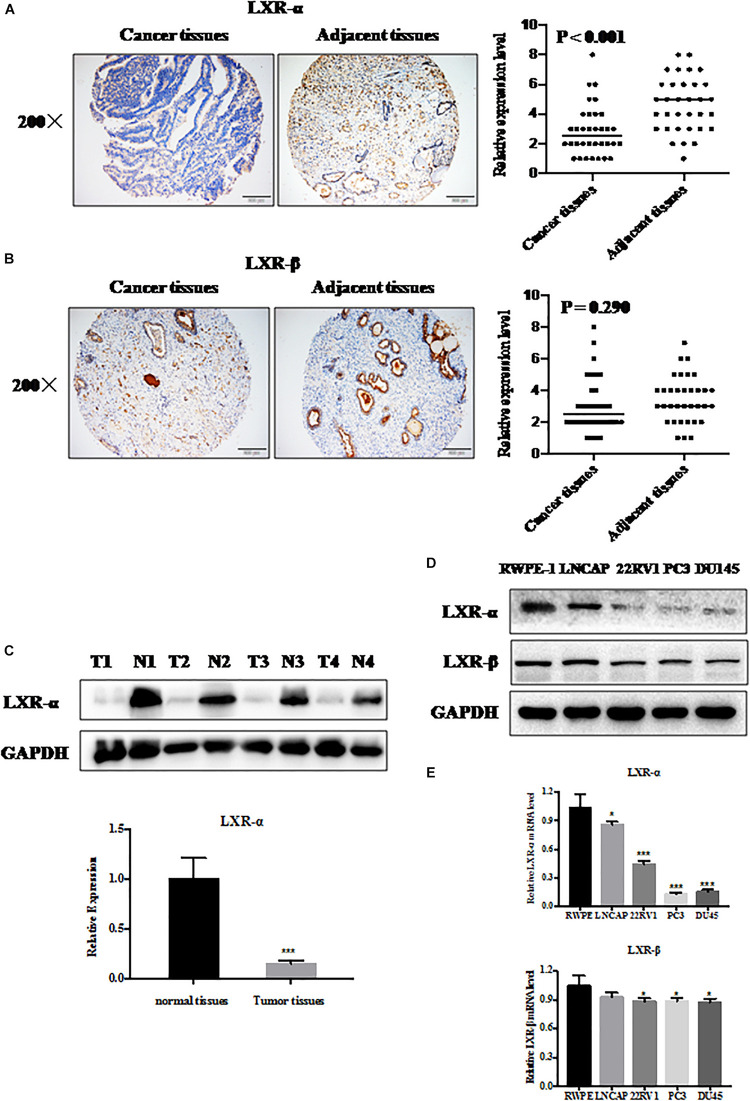
LXR-α is down-regulated in prostate cancer cells and tissues. **(A,B)** LXR-α and LXR-β proteins expressed in prostate cancer tissues and adjacent tissues (*n* = 34) by immunohistochemistry (IHC). The microscope image was taken at ×200 magnification. **(C)** LXR-α protein levels in prostate cancer tissues and matched precancerous tissues were analyzed by IB analysis. **(D,E)** The levels of LXR-α and LXR-β in the prostate cells were analyzed by immunoblotting (IB) analysis. Data were presented as means ± SD. *p < 0.05, ***p < 0.001, compared with the control group; *n* = 3. GAPDH levels served as the control for equal loading.

### Bioinformatics Analysis

Gene-Cloud Biotechnology information (GCBI)^1^ is an online comprehensive bioinformatics analysis platform that predicts the regulation of genes and ncRNAs, transcription factors, and gene expression levels in disease. In the present study, GCBI was used to identify transcriptional regulators of LXR-α.

### Statistical Analysis

All data were shown as the average ± standard deviation (Mean ± SD) and each experiment was repeated at least three times independently. All statistical analyses were performed using the GraphPad Prism Version 5.0 (San Diego, CA, United States). The statistical significance of differences between groups was examined by one-way analysis of variance (ANOVA) followed by Tukey’s multiple comparison procedure or student’s *t*-test. A *p*-value of less than 0.05 was considered statistically significant. Both CalcuSyn software and Jin’s formula were used to evaluate the synergistic effects of drug combinations as described previously (41, 42).

## Results

### LXRs Express in Prostate Cancer Cells and Tissues

To evaluate the expression of LXRs in prostate cancer tissue and normal tissue, we measured their expression status by immunoblotting (IB), immunohistochemistry (IHC), and q-PCR analysis. We selected 34 pairs of prostate cancer tissues and adjacent normal tissues from the tissue microarray. We found that LXR-α (Figure 1A), but not LXR-β (Figure 1B) protein level, was significantly reduced in prostate cancer tissues compared with adjacent normal tissues. We also collected 4 pairs of clinical samples (including tumor tissues and adjacent normal tissues) to detect the protein expression level of LXR-α and further confirmed that LXR-α protein level in prostate tumor tissue was lower than that in matching normal tissue (Figure 1C). Among LNCaP, 22RV1, PC3, and DU145 prostate cancer cell lines and one immortalized cell line (RWPE-1) tested, mRNA and protein levels of LXR-α were lower in prostate cancer cell lines (Figures 1D,E), whereas mRNA and protein levels of LXR-β were similar in all cell lines (Figures 1D,E).

### LXR-α Inhibits Prostate Cancer Progression and Metastasis

**FIGURE 2:**
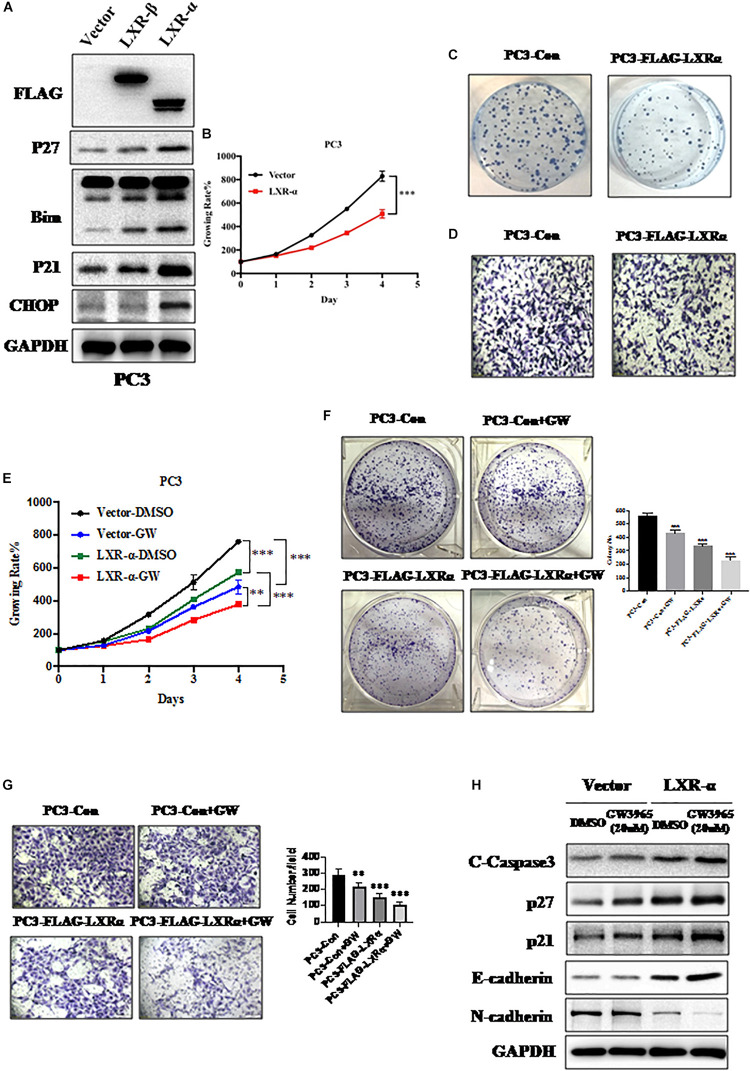
Activation of LXR-α inhibits prostate cancer progression and metastasis. **(A)** Effects of LXRs’ overexpression on the expression of cell progression-related protein in PC3 cell line. **(B)** Overexpression of LXR-α significantly inhibited cell proliferation in the PC3 cell line. Cell viability was detected with Cell Counting Kit-8 (CCK-8) assay. **(C)** Overexpression of LXR-α significantly suppressed colony formation in the PC3 cell line. **(D)** Overexpression of LXR-α extremely decreased cell invasion ability of the PC3 cell line. **(E)** Compared with LXR-α transfected alone, co-treatment with GW3965 could better inhibit cell proliferation. Cell viability was detected with CCK-8 assay. **(F)** Overexpression of LXR-α with concomitant treatment with GW3965 resulted in a greater inhibition of the clone-forming ability of PC3 cell line than GW3965 alone. **(G)** LXR-α overexpression decreased cell invasion ability of PC3 cell line, and did so more significantly through co-treatment with GW3965. **(H)** Compared with LXR-α transfected alone, co-treatment with GW3965 could better increase p21, p27, E-cadherin, and C-Caspase 3. Data were presented as means ± SD. *^∗∗^p* < 0.01, *^∗∗∗^p* < 0.001, compared with the control group; *n* = 3. GAPDH levels served as the control for equal loading.

To clarify the effect of LXRs on cell proliferation and metastasis of prostate cancer, we measured the levels of a few tumor suppressive and pro-apoptotic proteins upon LXRs overexpression. We found that overexpression of LXR-α induces the accumulation of multiple tumor suppressor proteins, including p21 and p27 (Figure 2A), which are known to regulate cell growth. Similarly, CHOP and Bim, as pro-apoptotic proteins, increased when LXR-α was overexpressed (Figure 2A). Compared to LXR-α overexpression, LXR-β overexpression had less influence on tumor suppressive and pro-apoptotic proteins (Figure 2A). Therefore, we selected LXR-α as the experimental subject. Next, we found that overexpression of LXR-α significantly inhibited cell growth (Figure 2B), clone-forming ability (Figure 2C)m and invasion ability (Figure 2D) of the PC3 cell line, suggesting that LXR-α was involved in prostate cancer progression and metastasis. LXRs are ligand-dependent receptors, thus, we next tested the effect of the GW3965 on the biological function of prostate cancer cells. GW3965 is a liver X receptor (LXR) agonist, which needs to be combined with LXR expression to exert its effect. The higher the expression level of LXR, the better the effect of GW3965. Compared with LXR-α transfected alone, co-treatment with GW3965 showed a better inhibition of cell proliferation (Figure 2E), colony-formation (Figure 2F), and invasion (Figure 2G) of the PC3 cell line with increased expression of p21, p27, E-cadherin, and C-Caspase 3, but decreased expression of N-cadherin (Figure 2H). These data suggested that a simultaneous increase of LXR-α can help to further inhibit progression and metastasis of prostate cancer.

**FIGURE 3:**
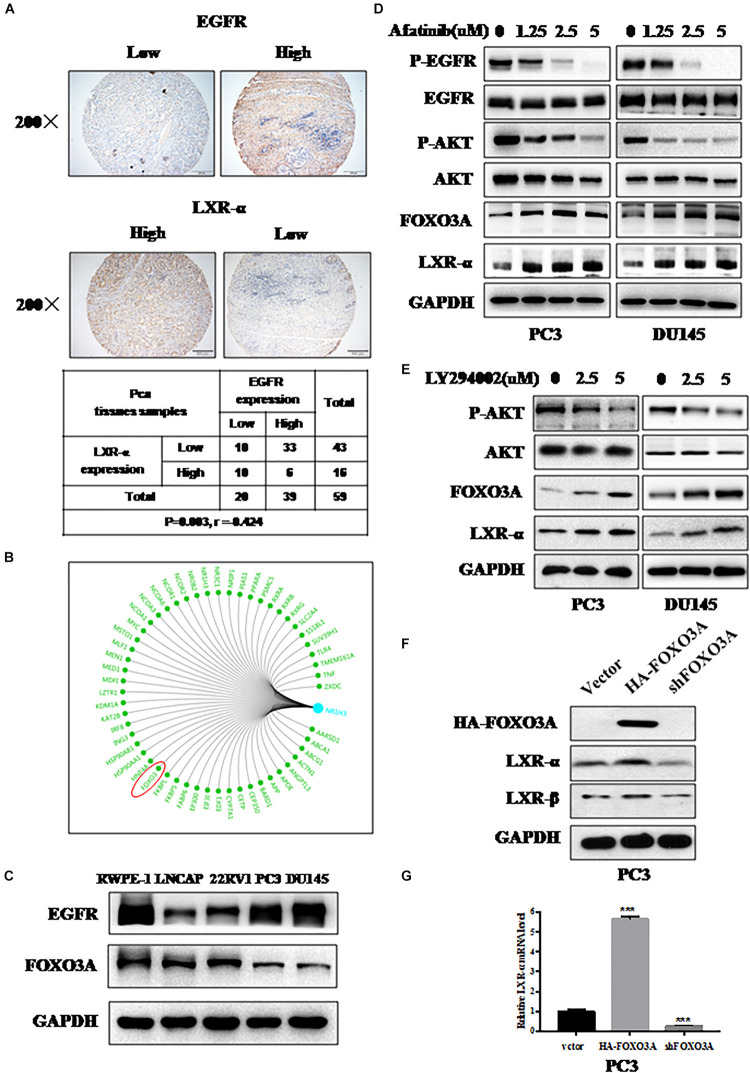
LXR-α is regulated by EGFR/AKT/FOXO3 pathway. **(A)** LXR-α and EGFR protein expressed in prostate cancer (*n* = 59) by IHC. The microscope image was taken at ×200 magnification. **(B)** Bioinformatics analysis comes from https://www.gcbi.com.cn/gclib/html/index. **(C)** Basal level of FOXO3A and EGFR protein in prostate cell lines. **(D)** EGFR inhibitor Afatinib dose-dependently increased FOXO3A and LXR-α levels in PC3 and DU145 cell lines. Prostate cancer cell lines were treated with different doses of Afatinib for 48 h, followed by determination of related-protein expression using IB analysis. **(E)** AKT inhibitor LY294002 dose-dependently increased FOXO3A and LXR-α levels in PC3 and DU145 cell lines. Prostate cancer cell lines were treated with different doses of LY294002 for 48 h, followed by determination of related-protein expression using IB analysis. **(F)** In a transfection experiment, the levels of endogenously expressed LXR-α were increased upon FOXO3A transfection, while they were reduced by FOXO3A knockdown. **(G)** Overexpression of FOXO3A elevated the level of LXR-α mRNA, while they were reduced by FOXO3A knockdown. Data were presented as means ± SD. ***p < 0.001, compared with the control group; *n* = 3. GAPDH levels served as the control for equal loading.

### LXR-α Is Regulated by EGFR/AKT/FOXO3 Pathway

We found that LXR-α expression is lower in prostate cancer cell lines and tissues (Figure 1). Meanwhile, EGFR was overexpressed and associated with poor prognosis in prostate cancer (43), and oncogenic transformation by EGFR increased the demand for cholesterol (36). Thus, we speculated that down-regulation of LXR-α might be mediated by EGFR. Next, we measured LXR-α and EGFR expression status in prostate cancer tissues by IHC staining. We selected 59 cancer tissues from the tissue microarray and found that the high EGFR staining group is largely associated with the low staining group of LXR-α (*p* < 0.01, *r* = −0.424), and vice versa (Figure 3A). To further elucidate how LXR-α is down-regulated in prostate cancer cells, a genetic bioinformatics database, the GCBI, was searched, which provides a web-lab with bioinformatics approaches to manage large amounts of transcriptome results. We found 54 potential transcription factors that may regulate LXR-α (Figure 3B), including FOXO3A (Forkhead box-containing protein class O3a). Some papers reported that FOXO3A is a transcriptional regulator, which can be regulated by EGFR (44–46). We therefore measured FOXO3A and EGFR expression in prostate cancer cell lines by IB. We found that FOXO3A is less expressed in high-grade malignant tumor cells and is negatively correlated with EGFR expression (Figure 3C). We speculated that down-regulation of LXR-α might be mediated by EGFR/FOXO3A. Consistently, EGFR inhibitor Afatinib decreased p-EGFR and p-AKT on PC3 and DU145 prostate cancer cell lines, in a dose-dependent manner (Figure 3D). Meanwhile, Afatinib increased FOXO3A protein as well as LXR-α protein (Figure 3D). Similarly, AKT inhibitor LY294002 increased FOXO3A and LXR-α protein in PC3 and DU145 cell lines (Figure 3E). We then determined whether down-regulation of LXR-α might be mediated by EGFR/AKT/FOXO3A. In a transfection experiment, the levels of endogenously expressed LXR-α were increased upon FOXO3A transfection (Figure 3F), while reduced by FOXO3A knockdown. Likewise, overexpression of FOXO3A elevated the level of LXR-α mRNA, and vice versa (Figure 3G), suggesting that FOXO3A regulated LXR-α by transcriptional control. Collectively, our results support that LXR-α is regulated by the EGFR/AKT/FOXO3A pathway.

### Combination of GW3965 and Afatinib Shows Synergistic Lethality *in vitro*

**FIGURE 4:**
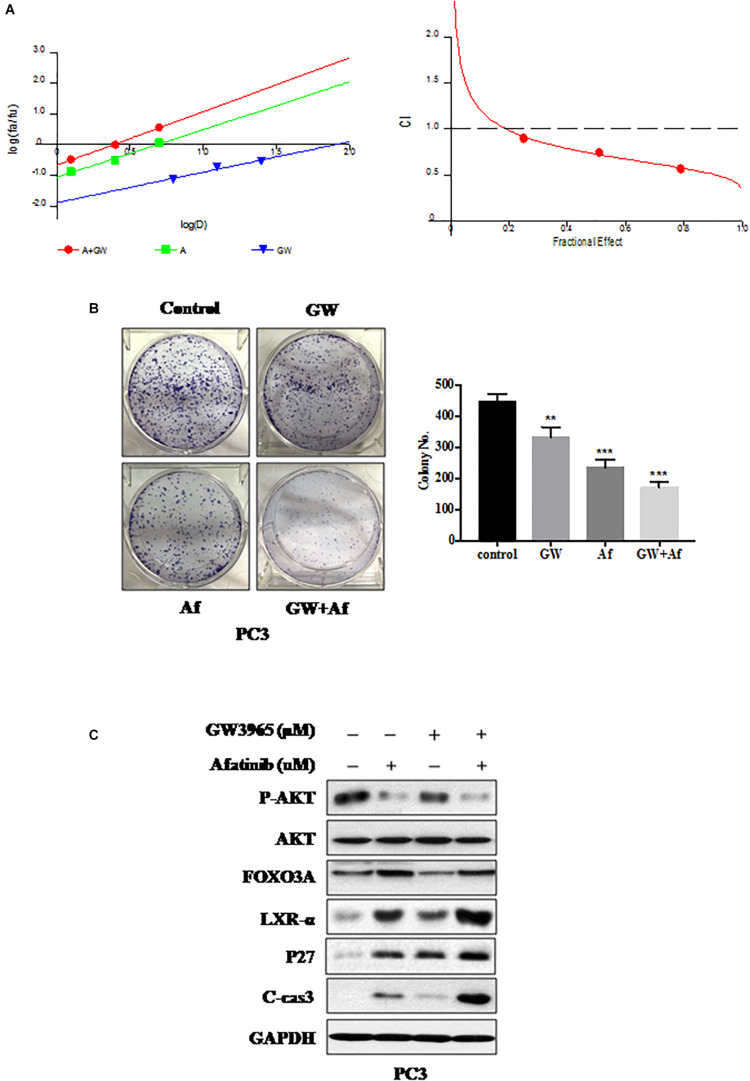
Combination of GW3965 and Afatinib shows synergistic lethality *in vitro*. **(A)** GW3965 and Afatinib showed synergistic effects in PC3 cell line. CI-effect plots and median effect plots were generated using CalcuSyn software. The points a, b, and c represent CI values for the combinations 5, 10, and 20 μM GW3965 with 1.25, 2.5, and 5 μM Afatinib in a constant ratio, respectively. **(B)** Combination of GW3965 and Afatinib inhibits cell colony formation in prostate cancer cells. PC3 cell line was treated with GW3965 (10 μM) or Afatinib (2.5 μM) alone or with a combination of the two compounds, followed by the colorogenic assay. The data were expressed as mean ± S.D. (**p < 0.01, ***p < 0.001; Student’s *t*-test) compared with control groups. **(C)** Combination of GW3965 and Afatinib simultaneously increased and activated LXR-α in the PC3 cell line. PC3 cell line was examined after 48 h of treatment with DMSO, 10 μM GW3965, and/or 2.5 μM Afatinib, and determined the related protein expression by IB analysis.

We found that the EGFR inhibitor Afatinib could increase LXR-α levels (Figure 3D) and LXRs agonist GW3965 could activate LXR-α (Figure 2). Therefore, we supposed that a combination of GW3965 and Afatinib should have dual inhibition effects on prostate cancer cells by increasing and activating LXR-α. Both CalcuSyn software and Jin’s formula were used as previously described to determine the synergy of the two agents. PC3 cell line was cultured with the combination of two drugs at different doses but in a constant ratio (GW3965 to Afatinib: 5–1.25 μM, 10–2.5 μM, and 20–5.0 μM, respectively) for 48 h. The combination of 5 μM GW3965 with 1.25 μM Afatinib in PC3 cell line inhibited cell proliferation by 28.0%, compared with monotherapy of GW3965 by 10.2% or Afatinib by 15.7%, indicating synergism (CI = 0.947; *Q* = 0.94; Figure 4A). Escalating doses, i.e., co-treatment with 10 μM GW3965 and 2.5 μM Afatinib (CI = 0.618; *Q* = 1.18) or 20 μM GW3965 and 5 μM Afatinib (CI = 0.538; *Q* = 1.29), showed synergetic effects in PC3 cell lines (Figure 4A). Furthermore, a combination of GW3965 with Afatinib significantly inhibited the clonogenic survival in the PC3 cell line (GW3965 or Afatinib vs. GW3965 + Afatinib: *P* < 0.01; Figure 4B), indicating that the combination of the two agents significantly inhibits cell proliferation or growth, which was further demonstrated by the increase of p27 (Figure 4C). In addition, the nature of suppression in cell proliferation or growth upon GW3965 and Afatinib combination was via induction of apoptosis (Figure 4C), as evidenced by the dose-dependent increased cleavage of caspase-3.

### Combination of GW3965 and Afatinib Shows Synergistic Lethality *in vivo*

**FIGURE 5:**
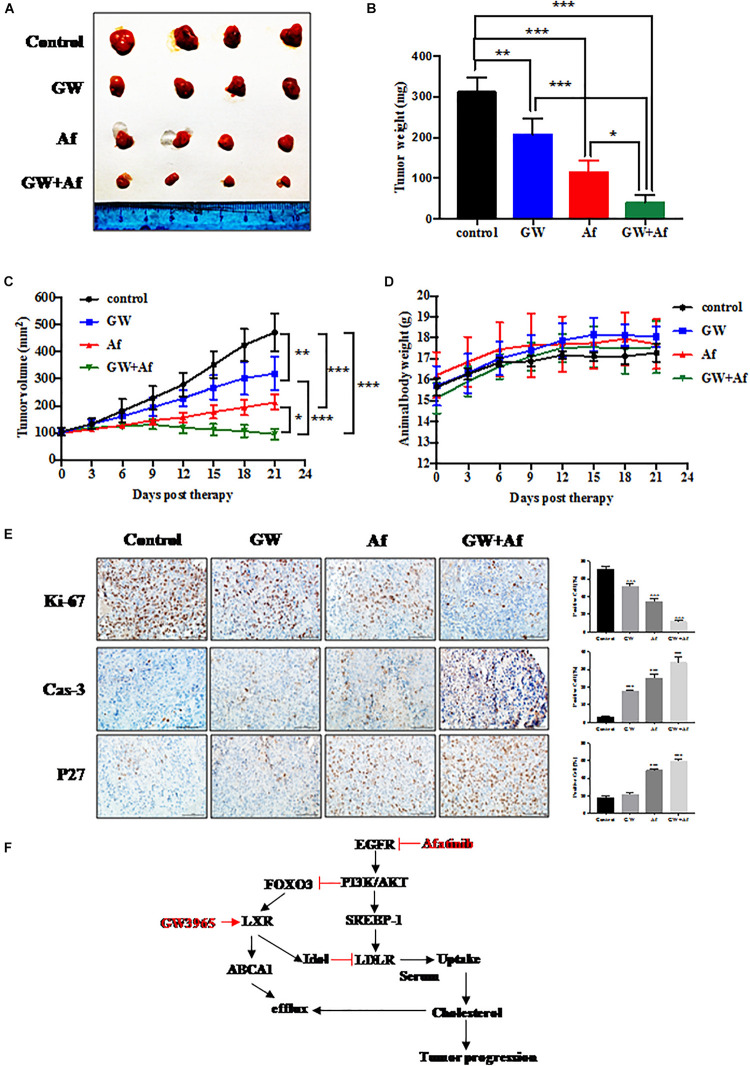
Combination of GW3965 and Afatinib shows synergistic lethality *in vivo*. **(A–E)** Synergistic anti-tumor activity of GW3965 and Afatinib in the PC3 xenograft model. PC3 cell line was injected subcutaneously into the right flank side of nude mice. The mice were randomized when the tumor size reached about 100 mm^3^ and were treated as follows: vehicle, *n* = 4; GW3965 (40 mg/kg/days for every day for 4 weeks), *n* = 4; Afatinib (20 mg/kg/days for every day for 4 weeks), *n* = 4; GW3965 + Afatinib, *n* = 5. The tumor growth was monitored and growth curve was plotted **(C)** and tumors were harvested **(A)** and weighted **(B)**. Body weight was measured during the treatment and plotted **(D)**. Schematic model depicting the mechanisms of action underlying nobiletin-induced anti-proliferation. **(E)** Immunohistochemical staining of xenograft tumor tissues. Tumor tissues from four groups of mice were fixed, sectioned, and stained with indicated antibodies. Scale bars: 100 μm. Shown are mean ± SEM, *p < 0.05; **p < 0.01, ***p < 0.001. **(F)** Schematic model depicting the mechanisms of action underlying LXR-α inhibited prostate cancer progression, which was regulated by EGFR/AKT/FOXO3 pathway.

We next validated the above *in vitro* findings by using an *in vivo* xenograft model. PC3 xenograft model was established and treated with vehicle, GW3965, and/or Afatinib. Consistent with the *in vitro* results, the combination of GW3965 and Afatinib suppressed tumor growth significantly more than single-agent treatment (Figure 5A). Consistently, the average tumor size and tumor weight at the end of experiment (treatment with 21 days) were significantly lower in the combination GW3965 and Afatinib group (Figures 5B,C). Body weight of the xenograft model was unchanged during drug treatment, suggesting that the effect on normal tissues was minimal (Figure 5D). Importantly, IHC staining of tumor tissues revealed that, compared with GW3965 or Afatinib single-agent treatment, a combination of the two agents more significantly inhibited cell growth (decrease of Ki-67 and increase of p27) and induced apoptosis (increase of cleavage caspase-3) (GW3965 or Afatinib vs. GW3965 + Afatinib: *P* < 0.01; Figure 5E). Collectively, the combination of GW3965 with Afatinib more significantly inhibited prostate cell growth and survival than single-agent treatment, with less effect on normal tissues.

## Discussion

Liver X receptors, including LXR-α and LXR-β, are ligand-dependent nuclear receptors, which have more anti-proliferative effects on a variety of cancer cells (28–31). Our results demonstrated that LXR-α had more anti-proliferative effects on prostate cancer cell lines than LXR-β (Figures 1, 2). We also revealed a positive correlation between lower LXR-α levels and high-grade malignancy prostate cancer cell lines, and vice versa (Figures 1D,E). LXR-α has been implicated in growth regulation of a variety of cancer cells (28, 47, 48), but the regulation of prostate cancer cell growth has never been previously reported. Herein, we showed that LXR-α acts as a tumor suppressor in prostate cancer with the following lines of supporting evidence: (1) LXR-α is down-regulated in prostate cancer tissues and high levels of LXR-α predicts a biomarker for prostate cancer; (2) Overexpression of LXR-α significantly inhibits the growth and metastasis of prostate cancer cells; and (3) The tumor suppressor function of LXR-α is mediated by the accumulation of the bona fide tumor suppressors, including p21 and p27. Some papers reported activation of LXRs could increase p21 and p27 (32, 33, 49). LXR agonists have been developed as potential drugs for the treatment of cardiovascular diseases and metabolic syndromes (50, 51), which are potential targets in cancer prevention and treatment. Previous studies demonstrated that LXR agonist T0901317 inhibited prostate cancer cell growth (32, 33), while our results further demonstrated the LXR agonist GW3965 can inhibit growth and metastasis of prostate cancer cells (Figure 2).

Although LXR-α inhibits cancer cell growth, the underlying mechanisms of this have never been previously reported. We found, for the first time, that LXR-α is regulated by the EGFR/AKT/FOXO3A pathway. Our conclusion is supported by the following lines of evidence: (1) Among 59 tumor TMA samples, the high EGFR staining group is largely associated with the low staining group of LXR-α, and vice versa; (2) Among prostate cell lines tested, there is a general tendency of inversed protein levels between EGFR and FOXO3A. FOXO3A could induce cholesterol regulation and lipid management (52), and be regulated by EGFR (44–46); (3) EGFR inhibitor Afatinib inhibited p-AKT and increased FOXO3A and LXR-α, as did AKT inhibitor LY294002; (4) The levels of endogenously expressed LXR-α were increased upon FOXO3A transfection, while they were reduced by FOXO3A knockdown; and (5) Overexpression of FOXO3A elevated the level of LXR-α mRNA, and vice versa.

Afatinib shows potent activity against wild-type and mutant forms of EGFR (53–56). However, patients who initially benefit from EGFR-targeted therapies eventually develop resistance (19). Afatinib had limited anti-tumor activity in unselected advanced CRPC patients (18). Thus, innovative treatment strategies are urgently needed for improving the survival of patients with prostate cancer. We found a combination of GW3965 and Afatinib simultaneously increased and activated LXR-α, which led to increased expression levels of tumor suppressor p27, eventually inhibiting tumor formation. Recently, it was shown that EGFR inhibitor synergized with LXRα agonists in killing cancer cells (36); for example, LXR ligands combined with gefitinib could better suppress cell cycle progression in NSCLC cells (57, 58). Therefore, a combination of LXR agonist GW3965 and Afatinib may serve as a potential therapeutic strategy for prostate cancer.

Our study also raised a question. It is well known that EGFR/PI3K/Akt signaling regulates LDLR mediated by SREBP-1, thereby promoting cell uptake of cholesterol from serum (35). Herein, we only confirmed that the EGFR/AKT signaling pathway can also down-regulate the expression of LXR-α by inhibiting the expression of the transcription factor FOXO3A. We will continue to pursue whether EGFR disturbs cancer cell cholesterol homeostasis by down-regulating cholesterol efflux through this new signal pathway, so as to have a more comprehensive understanding of the effect of EGFR on the metabolism of cholesterol in cancer cells.

## Conclusion

In conclusion, we reveal that the protein level of LXR-α, but not LXR-β, is higher in prostate cancer tissues than adjacent normal tissues. Moreover, we find that LXR-α is regulated by the EGFR/AKT/FOXO3A pathway. Afatinib, an EGFR inhibitor, down-regulates the activity of PI3K/AKT signaling pathway by inhibiting EGFR, thereby promoting the expression of FOXO3A. FOXO3A as a transcription factor further up-regulates the expression of LXR-α. GW3965 is a liver X receptor (LXR) agonist, which needs to be combined with LXR to exert its effect. Therefore, we further confirm that a combination of Afatinib and GW3965 simultaneously increases and activates LXR-α, resulting in an increase of tumor suppressors and, ultimately, tumor inhibition (Figure 5F). Above all, this combination therapy may become a potential treatment strategy for prostate cancer, especially progressive prostate cancer.

## Data Availability Statement

The raw data supporting the conclusions of this article will be made available by the authors, without undue reservation.

## Ethics Statement

The studies involving human participants were reviewed and approved by Ethics Committee of the Army Medical University (Third Military Medical University) of China. The patients/participants provided their written informed consent to participate in this study. The animal study was reviewed and approved by the Institutional Animal Care and Use Committee of Model Animal Research Center of Army Medical University (Third Military Medical University) of China.

## Author Contributions

WF and JX did the conception and design of the research, and edited and revised the manuscript. TC performed the experiments, interpreted the results of the experiments, and prepared the figures. TC and WF drafted the manuscript. All authors read and approved the final manuscript.

## Conflict of Interest

The authors declare that the research was conducted in the absence of any commercial or financial relationships that could be construed as a potential conflict of interest.

## Footnotes

1 http://www.gcbi.com.cn/gclib/html/index
